# Dual RNA-Seq of Trunk Kidneys Extracted From Channel Catfish Infected With *Yersinia ruckeri* Reveals Novel Insights Into Host-Pathogen Interactions

**DOI:** 10.3389/fimmu.2021.775708

**Published:** 2021-12-15

**Authors:** Yibin Yang, Xia Zhu, Haixin Zhang, Yuhua Chen, Yi Song, Xiaohui Ai

**Affiliations:** ^1^ Yangtze River Fisheries Research Institute, Chinese Academy of Fishery Sciences, Wuhan, China; ^2^ The Key Laboratory for Quality and Safety Control of Aquatic Products, Ministry of Agriculture, Beijing, China; ^3^ Fish Disease Laboratory, Jiangxi Fisheries Research Institute, Nanchang, China; ^4^ Department of Gastroenterology, Zhongnan Hospital of Wuhan University, Wuhan, China; ^5^ Hubei Clinical Center & Key Lab of Intestinal & Colorectal Diseases, Zhongnan Hospital of Wuhan University, Wuhan, China

**Keywords:** channel catfish, *Yersinia ruckeri*, dual RNA-seq, host-pathogen interactions, immunity, virulence

## Abstract

Host-pathogen intectarions are complex, involving large dynamic changes in gene expression through the process of infection. These interactions are essential for understanding anti-infective immunity as well as pathogenesis. In this study, the host-pathogen interaction was analyzed using a model of acute infection where channel catfish were infected with *Yersinia ruckeri*. The infected fish showed signs of body surface hyperemia as well as hyperemia and swelling in the trunk kidney. Double RNA sequencing was performed on trunk kidneys extracted from infected channel catfish and transcriptome data was compared with data from uninfected trunk kidneys. Results revealed that the host-pathogen interaction was dynamically regulated and that the host-pathogen transcriptome fluctuated during infection. More specifically, these data revealed that the expression levels of immune genes involved in Cytokine-cytokine receptor interactions, the NF-kappa B signaling pathway, the JAK-STAT signaling pathway, Toll-like receptor signaling and other immune-related pathways were significantly upregulated. *Y. ruckeri* mainly promote pathogenesis through the flagellum gene fli*C* in channel catfish. The weighted gene co-expression network analysis (WGCNA) R package was used to reveal that the infection of catfish is closely related to metabolic pathways. This study contributes to the understanding of the host-pathogen interaction between channel catfish and Y. ruckeri, more specifically how catfish respond to infection through a transcriptional perspective and how this infection leads to enteric red mouth disease (ERM) in these fish.

## Introduction

Aquaculture is one of the most dynamic sectors in the global food system and has made significant contributions to the production of protein rich food consumed by humans (1). The global aquaculture industry has grown by 5% annually over the past 20 years (2, 3). However, more recently, the practice of aquaculture has encountered many challenges, one being disease outbreak caused by microbial pathogens (4). Channel catfish (*Ictalurus punctatus*) were introduced to China from the United States in 1984. After adapting to the environment in China, there was an explosive growth rate in the breeding yield and area. Channel catfish are a major component of freshwater fish culture in China. However, disease in these fish lead to a bottleneck restricting further growth of the channel catfish industry. The main pathogenic microorganisms infecting channel catfish include *Aeromonas hydrophila*, *Yersinia ruckeri*, *Edwardsiella ictaluri* and *Streptococcus iniae*, which have resulted in great economic loss for the aquaculture industry (4–10). *Y. ruckeri* is a gram-negative bacterium and the cause of enteric red mouth disease (ERM) in fish (11). ERM is a serious disease prevalent in aquaculture worldwide and is commonly observed in rainbow trout, sturgeon and channel catfish (12–16).

Sick channel catfish show sepsis and inflammation in most organs, including the trunk kidney, spleen, liver and gastrointestinal tract (6). During the early stage of infection, the pathogen may invade into the gills and gastrointestinal epitheliums. Later, the infection may enter the blood circulation system and infect the spleen and trunk kidney, accumulate in the lymphoid organs and finally destroy the immune system (17). *Y. ruckeri* is a facultative, intracellular pathogen that can survive in macrophages *in vitro* and *in vivo*. In fact, there is quite a bit more to understand about the physiology of *Y. ruckeri* since most research has been focused on single virulence factors, such as extracellular toxins, high affinity iron uptake systems and resistance to innate immune mechanisms (18–26). Liu et al. identified that Toll/IL-1 receptors containing the proteins stir-1, stir-2 and stir-3 synergistically assisted the immune escape of *Y. ruckeri* SC09 (27, 28). This study also found that the virulence protein of *Y. ruckeri* TIR mediated immune escape by targeting the MyD88 receptor (29). The molecular mechanisms behind the infection of channel catfish by *Y. ruckeri* is still not very clear.

Infection is a life and death struggle between the host and its pathogen (30). In this struggle, both the pathogen and host must mobilize all of their available resources to win (31). The transcriptomic profiles for both the host and pathogen vastly alter during the infection process (32, 33). Traditional RNA sequencing (RNA-seq) technology only reflects the host transcriptome (34) and does not detect the host and pathogen transcriptome simultaneously. However, dual RNA-seq can provide analysis of the interaction between the host and pathogen transcriptomes. Dual RNA-seq has been used to study a variety of bacterial infections, including cell and animal infection models and has identified many key genes related to host-pathogen interactions (35).

To better understand the interaction between channel catfish and *Y. ruckeri*, an infection model was established to analyze dual RNA-seq data of the trunk kidney at different stages of infection and to monitor changes in the transcriptomes of both channel catfish and *Y. ruckeri*. Results obtained during this study will better clarify the host-pathogen interaction between channel catfish and *Y. ruckeri*.

## Materials and Methods

### Ethics Statement

This study was performed strictly in accordance with the Guidelines for the Care and Use of Animals for scientific purposes formulated by the Institutional Animal Care and use Committee of Changjiang Fisheries Research Institute, Chinese Academy of Fishery Sciences.

### Maintenance and Treatment of Channel Catfish

All healthy and moderate size (100 ± 20 g) channel catfish were purchased from Wuhan Baishazhou aquatic market. These fish were domesticated for one week at a temperature of 25 ± 1°C under laboratory conditions free of specific pathogens before infection. The pathogenic *Y. ruckeri* YZ strain (GenBank accession number is OL376599) was isolated from infected channel catfish. *Y. ruckeri* was cultured on a nutrient agar plate at 28°C; for 18 h, washed with sterile PBS, blown evenly, diluted to 10^8^ CFU/mL and then inoculated in healthy channel catfish. A bacterial sample was also collected as a control. The experimental group was injected with 10^5^ CFU/g of *Y. ruckeri* through the base of channel catfish ventral fin, and 100 fishes were used in the experimental group and the control group respectively. The same volume of sterile phosphate buffered saline (PBS) was injected into the same area of the channel catfish for the control group. The water temperature was maintained at 25°C throughout the experiment. The dynamics of the catfish were observed and recorded every 6h, which swimming behavior, symptom, mortality and so on. The preliminary experiments had confirmed that channel catfish can be successfully infected by *Y. ruckeri* at 6,12,24 hour post-injection(hpi). For dual RNA-seq assays, the trunk kidneys of 15 infected channel catfish were sampled at 6, 12 and 24 hpi. Five trunk kidneys were mixed as one sample. PBS-treated channel catfish and *in vitro*cultured *Y. ruckeri* (28°C) were used as controls. These experimental samples were then fixed using Trizol to extract total RNA.

### RNA Isolation, cDNA Library Construction and Sequencing

Total RNA from trunk kidneys and *Y. ruckeri* were extracted using Trizol reagent (Invitrogen, USA). A turbo DNA free DNase (Abion, USA) treatment was performed to remove possible contaminated genomic DNA. RNA samples were further purified using the RNA zero RNA Removal Kit (human/mouse/rat or Gram-negative bacteria) (Epicentre, USA). Samples of mRNA were then fragmented and cDNA was synthesized using a superscript double stranded cDNA synthesis Kit (Invitrogen, USA). After modification and purification, the enriched library fragments were amplified using polymerase chain reaction (PCR). The library quality was analyzed using an Agilent 2100 biological analyzer (Agilent technology, USA). Second generation sequencing was performed using an Illumina novaseq sequencing platform.

### Illumina Sequencing, Data Processing and Quality Control

The obtained reads were cleared using fastqc software (http://www.bioinformatics.babraham.ac.uk/projects/fastqc/) and the content and quality of the remaining cleaning reads were evaluated. Low-quality reads and 3’ adapter sequences were removed using Trim Galore. A comparative analysis was then performed using the reference genome of channel catfish and *Y. ruckeri*. For each sample belonging to the experimental and control groups, TopHat was used to align the reference genome sequence (16, 36).

### Identification of Differentially Expressed Genes (DEGs)

To evaluate the transcript expression levels in the different groups, rsem software with default parameter settings was used to estimate the expression levels (relative abundance) of specific transcripts mapped per million fragments per thousand base transcripts (frkm) (37, 38). The expression levels of each transcript were transformed using base log_2_ (fpkm + 1). DESeq software was used to screen DEGs and calculate the fold changes of transcripts (39). Two-fold changes were considered for further study and a p value < 0.05 was considered statistically significant.

### GO Functional Annotation and Enrichment Analysis for DEGs

To analyze the potential functions of DEGs, we first annotated them as depicted on the uniprot database (http://www.uniprot.org/). Then, the DEGs were functionally annotated using gene ontology (GO) terms (http://www.geneontology.org) with the use of Blast2GO (https://www.blast2go.com/) (40). All DEGs were mapped to GO terms in the GO database and the number of genes for every term was calculated. A hypergeometric test was used to detect the enriched GO terms of the DEGs when compared with the transcriptome background, as follows:


$$p=1-\sum_{i=0}^{m-1}\frac{\left(\begin{array}{c} M\\ i \end{array}\right)\left(\begin{array}{c} N-M\\ n-i \end{array}\right)}{\left(\begin{array}{c} M\\ n \end{array}\right)}$$

In this formula, n represents the number of genes annotated with GO. N represents the number of DEGs in n, which is the number of genes mismatched with the annotation of specific GO terms. M is the number of DEGs. The calculated p value was subjected to Bonferroni correction. The corrected p value of 0.05 was determined as the threshold for statistical significance. When the corrected p value was < 0.05, the GO term was considered as significantly enriched.

### Pathway Analysis of DEGs

The pathway analysis for the DEGs was annotated using blastall (http://nebc.nox.ac.uk/bioinformatics/docs/blastall.html) against the yoto Encyclopedia of genes and genomes (KEGG) database. The enriched DEG pathways were determined using the same formula as used for GO analysis. In this formula, n represents the number of genes annotated using KEGG, N represents the number of DEGs in n, MRE represents the number of genes annotated by a specific pathway and M represents the number of DEGs.

### Weighted Gene Coexpression Network Analysis (WGCNA)

The WGCNA package in R was used to analyze the data (41). WGCNA helps identify functional pathways when studying interactions between species (42). The top 25% of genes were selected for WGCNA. A network was created based on results of the picksoftthreshold function. Hierarchical clustering and dynamic branch cutting were used to determine the stable module of tight junction genes. A hub gene was defined by module connectivity and measured by the absolute value of Pearson correlation. Hub genes in the module were considered to have functional significance. Key modules were visualized using Cytoscape (http://www.cytoscape.org/).

### Quantitative Real-Time Reverse-Transcription PCR (qRT-PCR)

qRT-PCR was used to confirm the expression levels of DEGs identified by RNA-seq analysis. Primers used are listed in **Table 1** (43–48). In channel catfish, EF-1 α was used as an endogenous control whereas gyrB was used for *Y. ruckeri*. qRT-PCR was performed using quantum Studio 6 flex (life technologies, USA) and melting curves were generated at the end of the run to confirm specificity. The 2^−ΔΔ^Ct method was used to calculate the relative levels of DEGs (49).

**Table 1:** Oligonucleotide primers used in qRT-PCR for DEG validation.

Species	Primers	Sequence (5′ to 3′)	Application
**Channel catfish**	col1a2-F	ATGGACGAAAAGGAGAGGCT	Real-time PCR
	col1a2-R	GGACCAGTGTTACCATCAGC	Real-time PCR
	ddx58-qF	ACCTGTTACCAGCCATCGTG	Real-time PCR
	ddx58-qR	TTGGGAGCTCCTCCGTAGAT	Real-time PCR
	egln3-qF	CCTCGGTGAAGCAATTGGTC	Real-time PCR
	egln3-qR	ATGGCTTCGGATCCTCTCTC	Real-time PCR
	trim35-qF	AGCGGCAGATTAAGGAGGAG	Real-time PCR
	trim35-qR	GTCTTCGGCTCTCATCTCCT	Real-time PCR
	agxt-qF	GAGAACGCGTGGCTGAAATC	Real-time PCR
	agxt-qR	ACTGAGGATTCTCCGTGTGC	Real-time PCR
	C5ar11-qF	CGCCAGCATCTTCACCCTGA	Real-time PCR
	C5ar11-qR	GCCATGGCACAGCATACCCA	Real-time PCR
	tnfaip3-qF	CACGCCTCGATGAGGGCAAT	Real-time PCR
	tnfaiP3-qR	GGGCGCCCATAGTGCATCTT	Real-time PCR
	IL-1β-qF	GTGTAAGCAGCAATCCAGTCA	Real-time PCR
	IL-1β-qR	CAAGCACAGAACAGTCAGGTAT	Real-time PCR
	Hsp70-qF	CTTGATGTTACCCCTCTGTCTCT	Real-time PCR
	Hsp70-qF	TCAGAGTAGGTGGTGAAAGTCTG	Real-time PCR
	EF-1α-qF	GTTGAAATGGTTCCTGGCAA	Real-time PCR
	EF-1α-qR	TCAACACTCTTGATGACACCAAC	Real-time PCR
***Y. ruckeri***	fliC-qF	CAGCGCTAAAGATGATGCAG	Real-time PCR
	fliC-qR	AATACCGTCGTTGGCGTTAC	Real-time PCR
	osmy-qF	CGGTTAGCGAATATGCCGGT	Real-time PCR
	osmy-qR	ACAAAACCACGCCATCGGTA	Real-time PCR
	flhD -qF	GATGGCTGATGCATTGTCTC	Real-time PCR
	flhD -qR	AGATCATCCACGCGAGATTC	Real-time PCR
	gyrB-qF	CGCTGGCCACTGTAAAGAAA	Real-time PCR
	gyrB-qR	GGCACTTTAACCGATACCACG	Real-time PCR

### Statistical Analysis

Data are expressed as mean ± standard deviation. SPSS 22 (American SPSS company) was used for data analysis. Independent sample t-tests, one-way analysis of variance (ANOVA) and Dunnett tests were used. A p value less than 0.05 were considered as statistically significant.

## Results

### Illumina Sequencing and Quality Assessment

The q20% and q30% of the sequencing data for each sample were between 96.01% ~ 98.63% and 89.32% ~ 96.22%, indicating that the overall sequencing quality was optimal and could be used for subsequent analysis (**Table 2**).

**Table 2:** Summary of reads obtained from dual RNA transcriptome sequencing.

Sample	Raw reads	Trimmed reads	Rate of clean Q20 bases (%)	Rate of clean Q30 bases (%)	GC content (%)	Trim rate (%)
**C1-1**	84,856,808	84,079,566	97.23	93.51	48.49	99.08
**C1-2**	81,247,528	80,495,852	97.41	93.81	48.73	99.07
**C1-3**	84,203,418	83,281,350	97.55	93.91	48.72	98.90
**C2-1**	7,350,026	6,644,382	98.59	96.10	48.21	90.40
**C2-2**	7,218,888	6,624,744	98.59	96.12	48.24	91.77
**C2-3**	8,767,566	8,009,270	98.63	96.22	48.31	91.35
**T6-1**	60,993,950	59,047,318	96.77	92.21	50.92	96.81
**T6-2**	65,274,668	63,394,046	96.57	91.83	51.16	97.12
**T6-3**	96,546,376	93,661,462	96.17	90.76	58.33	97.01
**T12-1**	104,731,406	101,790,432	96.01	90.39	60.10	97.20
**T12-2**	112,537,294	109,180,922	95.47	89.32	62.14	97.02
**T12-3**	104,156,370	102,205,876	97.44	93.62	60.92	98.13
**T24-1**	89,352,464	87,353,516	98.06	94.95	53.79	97.76
**T24-2**	138,930,384	136,266,932	97.52	93.69	61.47	98.08
**T24-3**	118,773,972	116,579,536	97.85	94.37	60.95	98.15

Q20 and q30 are the percentages of bases with phred values greater than 20 and 30 in the total bases respectively. C1 correspond to the catfish control and C2 correspond to the bacterial control.T6, T12 and T24 means 6, 12 and 24hpi Trunk kidney sample, and each experimental group consisted of three parallel.

### Comparison With the Reference Genome

Fish and bacterial samples were compared with channel catfish GCF_ 001660625.1 and *Y. ruckeri* jrwx01000000 reference genomes (**Tables 3** and **4**). The proportion of the two samples that could be compared to a single location was greater than 95% and the proportion of multiple locations was low, indicating that the sequencing quality was sufficient for further analysis.

**Table 3:** Statistical results for mapping the trimmed reads with the channel catfish reference genome.

Sample	Total reads	Unique mapped (%)	Multiple mapped (%)	Unmapped (%)
**C1-1**	84,079,566	92.11	3.31	4.58
**C1-2**	80,495,852	93.06	3.01	3.93
**C1-3**	83,281,350	93.17	2.94	3.89
**T6-1**	59,047,318	82.46	6.85	10.69
**T6-2**	63,394,046	81.44	6.72	11.84
**T6-3**	93,661,462	64.34	4.65	31.01
**T12-1**	101,790,432	53.42	4.52	42.06
**T12-2**	109,180,922	52.89	4.15	42.96
**T12-3**	102,205,876	57.60	3.84	38.56
**T24-1**	87,353,516	79.21	6.07	14.72
**T24-2**	136,266,932	56.97	3.91	39.12
**T24-3**	116,579,536	57.00	6.96	36.04

**Table 4:** Statistical results for mapping the trimmed reads with the *Y. ruckeri* reference genome.

Sample	Total reads	Unique mapped (%)	Multiple mapped (%)	Unmapped (%)
**C2-1**	6,644,382	86.79	0.65	12.56
**C2-2**	6,624,744	87.51	0.39	12.1
**C2-3**	8,009,270	87.68	0.48	11.84
**T6-1**	59,047,318	0.04	1.84	98.12
**T6-2**	63,394,046	0.04	2.03	97.93
**T6-3**	93,661,462	0.01	0.55	99.44
**T12-1**	101,790,432	0.17	5.26	94.57
**T12-2**	109,180,922	0.05	1.30	98.65
**T12-3**	102,205,876	0.01	0.27	99.72
**T24-1**	87,353,516	0.01	0.35	99.64
**T24-2**	136,266,932	0.01	0.43	99.56
**T24-3**	116,579,536	0.01	0.24	99.75

### DEG Analysis

**Figure 1:**
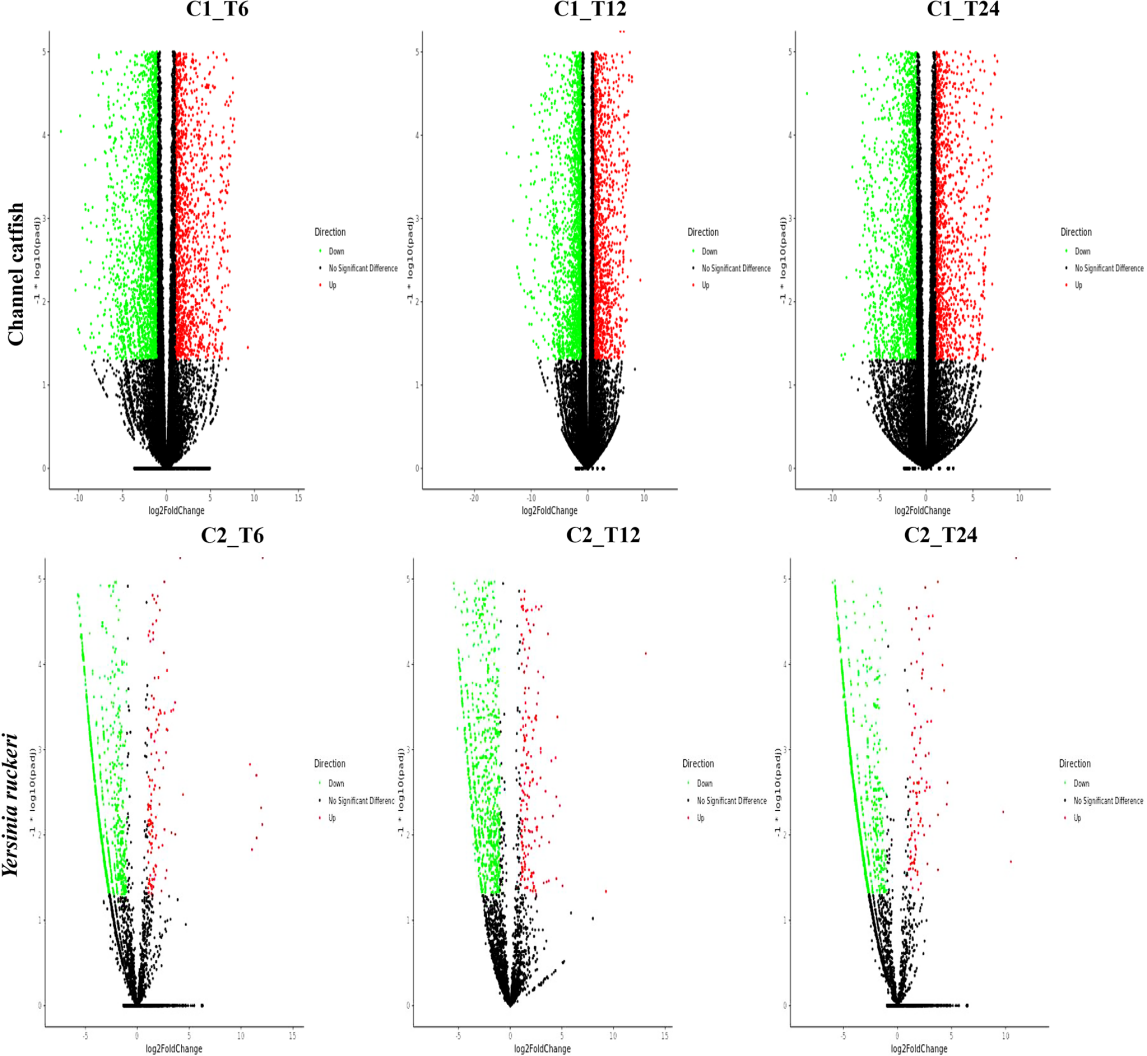
The gene expression profiles for channel catfish and *Y. ruckeri* during infection. A volcanic plot depicting the differences in the expression profiles of channel catfish and *Y. ruckeri*. The X-axis represents a log2 (fold change) and the Y-axis represents -log10 (p value). Red represents significantly upregulated genes whereas green represents significantly downregulated genes. Each dot represents a single gene. C1_T6(C2_T6) represents 6 hpi, C1_T12(C2_T12) represents the 12 hpi and C1_T24 (C2_T24) stands for 24 hpi.

**Figure 2:**
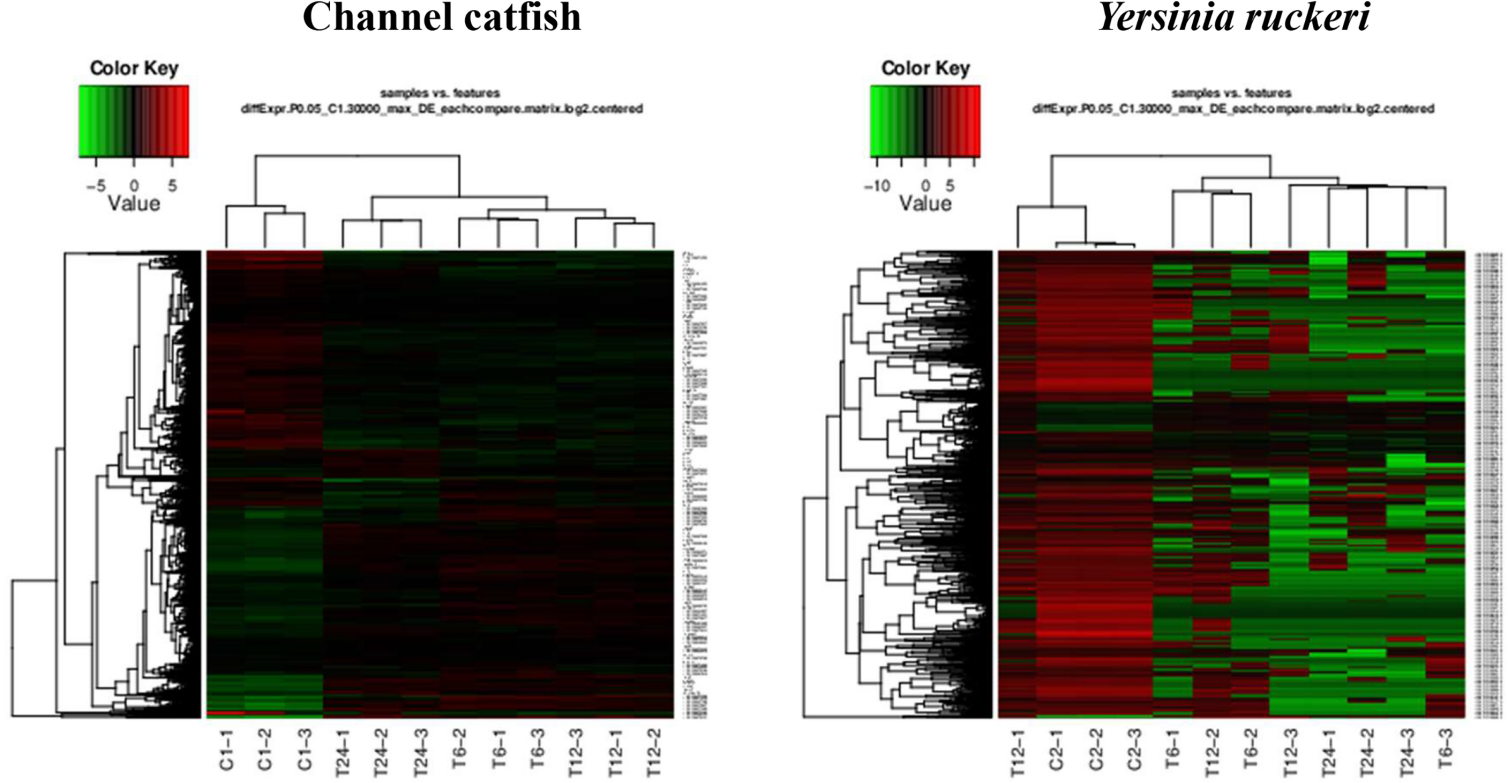
The gene expression profiles for channel catfish and *Y. ruckeri* using pattern clustering. Red represents the upregulated genes, green represents the downregulated genes. Each line represents a single gene. C1 and C2 as control group, T6, T12 and T24 represents the experimental group in 6 hpi, 12 hpi and 24 hpi.

To study and compare the DEGs between control fish and infected fish or the pathogen which infected fish and the pathogen before infection, a gene expression profile was generated using the cuffdiff program (**Figures 1** and **2**). Transcriptome sequencing revealed that 4628 DEGs were upregulated and4281 DEGs were downregulated at 6 hpi in the trunk kidney. At 12 hpi, the trunk kidney showed a significant increase in 4494 DEGs and decrease in 4463 DEGs. At 24 hpi, 4028 DEGs were upregulated and 4235 DEGs were downregulated in the trunk kidney. For *Y. ruckeri* a significant upregulation in 294 DEGs and downregulation in 994 DEGs were observed at 6 hpi. At 12 hpi, 346 DEGs were significantly upregulated and 975 DEGs were significantly downregulated in *Y. ruckeri*. At 24 hpi, 200 DEGs were upregulated and 1212 DEG were downregulated in *Y. ruckeri*. These results showed a strong interaction and reaction between the host and pathogen during the infection.

### GO Annotation of DEGs

**Figure 3:**
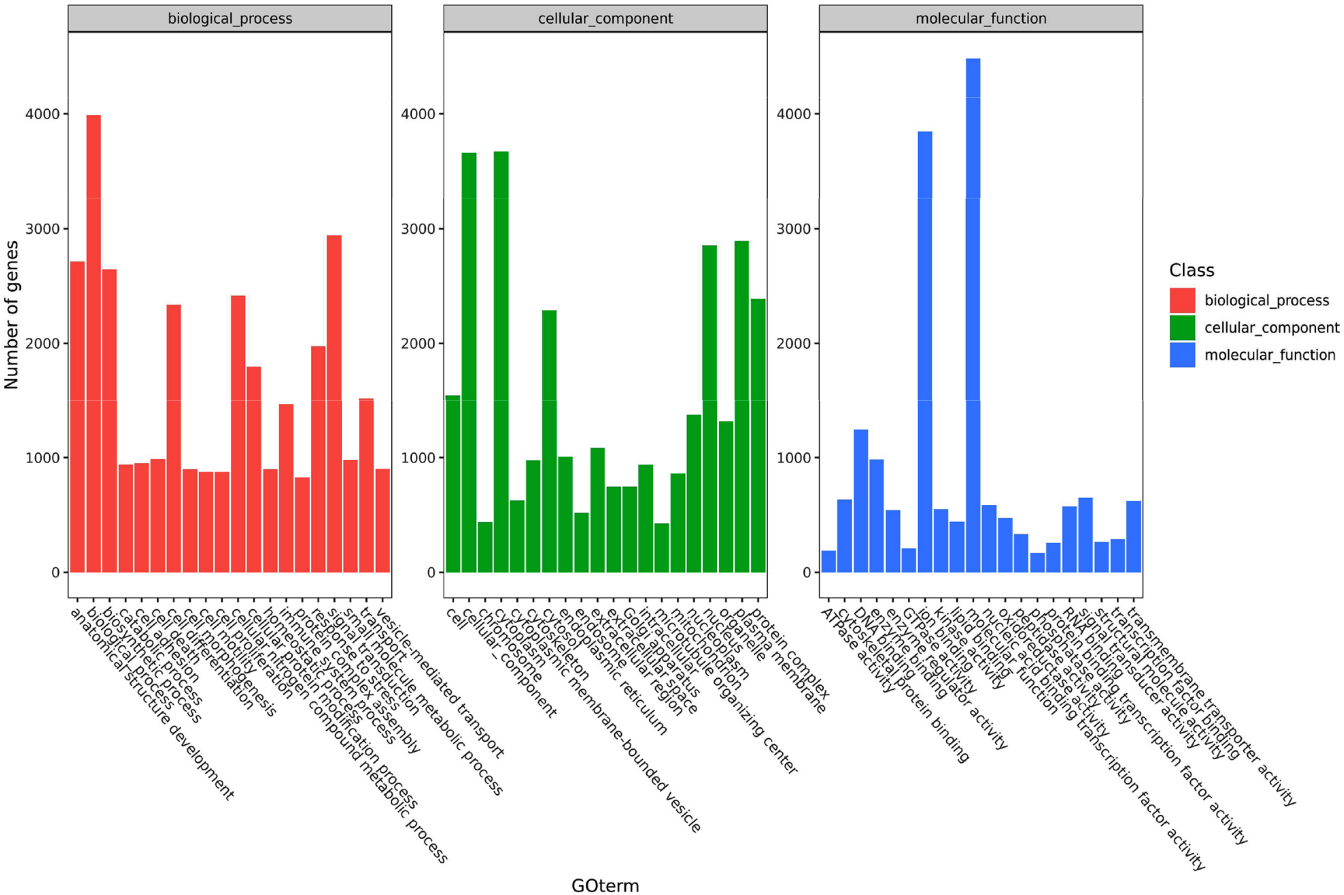
A histogram of the enriched subcategories after GO annotation of DEGs in channel catfish infected by bacteria. The GO terms (X-axis) were grouped into 3 main ontologies, including biological processes (BP), cellular components (CC) and molecular functions (MF). The Y-axis indicates the number of DEGs.

The functions of the DEGs in channel catfish are summarized in three groups, including cell components, molecular functions and biological processes. Secondary classifications of the top 20 GO terms with the most annotations under each classification are shown in **Figure 3**. The main subclasses for the biological processes category (containing a total of 3990 genes), includes signal transmission (2942 genes), analytical structure development (2714 genes), analytical structure development (2646 genes) and immune system (1465 genes). The main subclasses for the cell components category included the cytoplast (3673 genes), cellular components (3663 genes), plasma membrane (2891 genes) and nucleus (2853 genes). The main subclasses for the molecular functions category included ion binding (3847 genes), signal transducer activity (650 genes), DNA binding (1245 genes) and enzyme binding (982 genes) (**Figure 3**). Dividing the significantly upregulated and down-regulated DEGs at each time point and then performing GO analysis helps to dynamically compare the expression changes of the transcriptome at different time points. At 6 hpi, the up-regulated genes were significantly enriched in terms of the cell, cell parts, intracellular parts, binding, biological regulation and metabolic process categories. At 12 hpi, the up-regulated genes were significantly enriched in terms of intracellular parts, intracellular, binding, organelles, intracellular organelles, biological regulation and metabolic processes, signal transduction and regulation of cellular process. The up-regulated and down-regulated genes at 24 hpi were both significantly enriched in the cell, cell parts, cellular processes, organelles, metabolic processes, response to stimuli, biological regulation and other GO term categories.

**Figure 4:**
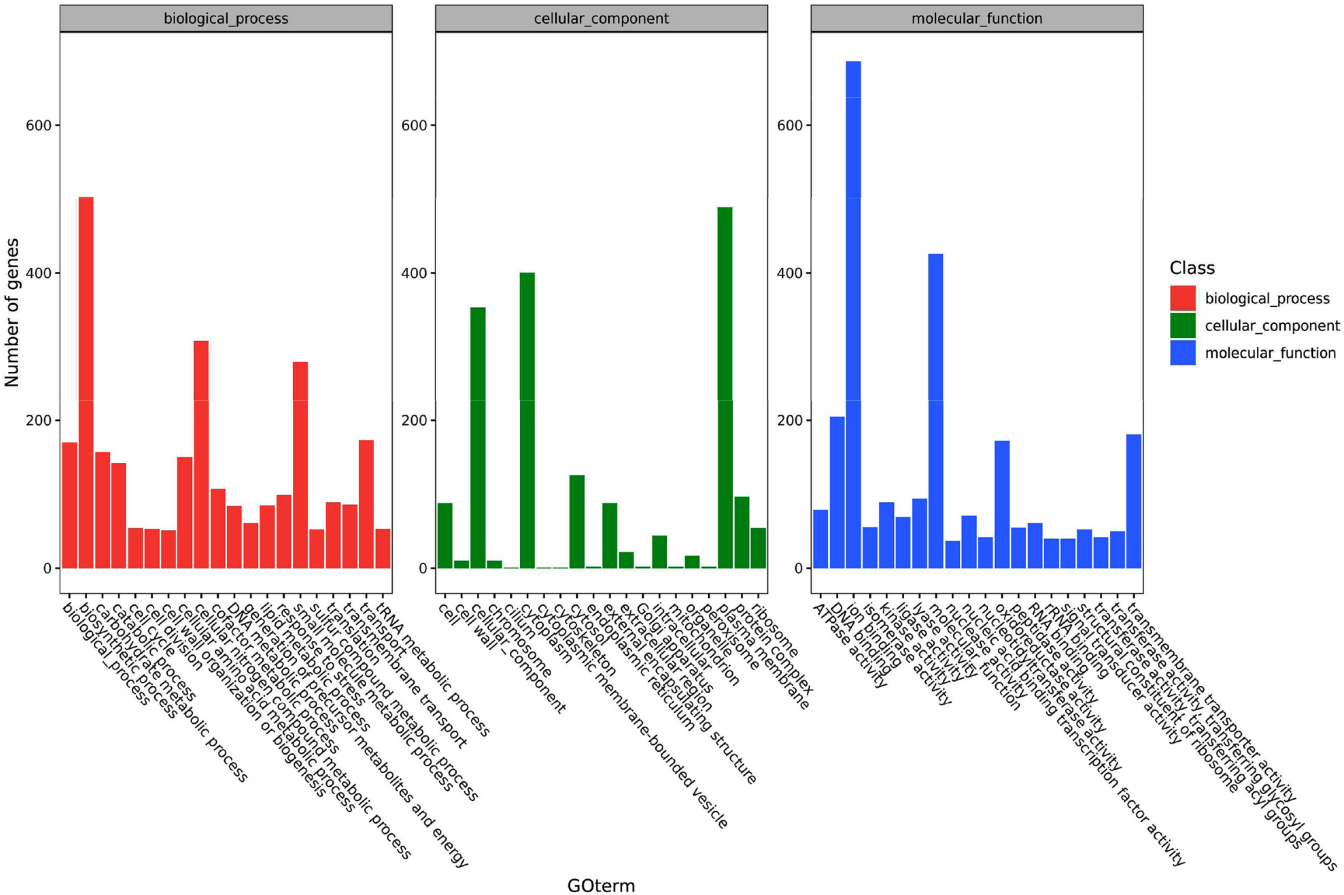
A histogram of the enriched subcategories after gene ontology (GO) annotation of the differentially expressed genes (DEGs) in *Y. ruckeri* at different time points. The GO terms (X-axis) were grouped into 3 main ontologies: biological process (BP), cellular component (CC), and molecular function (MF). The Y-axis indicates the number of DEGs.

The functions of the pathogen’s DEGs are summarized in three categories including cell components, molecular functions and biological processes. Secondary classifications of the top 20 GO terms were selected from the most annotated under each classification and are shown in **Figure 4**. The main subclasses of the biological processes included biosynthetic processes (503 genes), cellular nitrogen compound metabolic processes (308 genes), small molecular metabolic processes (280 genes) and transport (173 genes). The main subclasses of cell components were cellular components (353 genes), cytosol (126 genes), plasma membrane (489 genes) and the cytoplast (400 genes). The main subclasses of molecular functions were ion binding (687 genes), transmembrane transporter activity (181 genes), DNA binding (205 genes) and oxidoreductase activity (181 genes) (**Figure 4**). At 6 hpi, the up-regulated genes were significantly enriched in terms of the cell, cell parts, cellular processes and metabolic processes categories. At 12 hpi, up-regulated genes were significantly enriched in terms of the cell, cell parts, binding, cellular process and metabolic processes categories. The up-regulated and down-regulated genes at 24 hpi were both significantly enriched in GO terms such as the cell, cell parts, cellular processes and primary metallic processes.

### KEGG Pathway Analysis of DEGs

**Figure 5:**
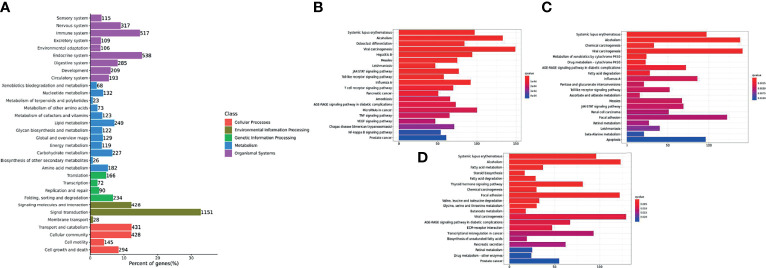
Histogram of the top 20 most-enriched KEGG pathways of DEGs in channel catfish infected by bacteria. The Y-axis represents KEGG pathway categories. The X-axis represents the statistical significance of enrichment. **(A)** represents the classification of total KEGG, **(B)** represents the top 20 KEGG enriched metabolic pathways in the C1_T6 group, **(C)** represents the top 20 KEGG enriched metabolic pathways in the C1_T12 group and **(D)** represents the top 20 KEGG enriched metabolic pathways in the C1_T24 group.

**Figure 6:**
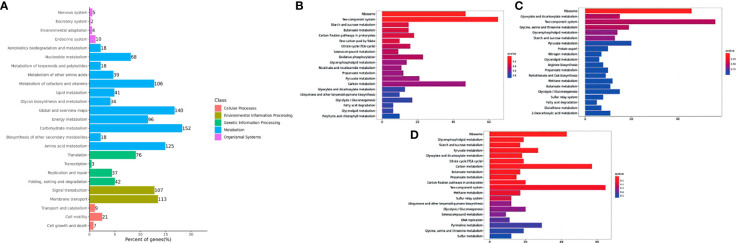
Histogram of the top 20 enriched KEGG pathways of DEGs in *Y. ruckeri* at different time points. The Y-axis represents the KEGG pathway categories. The X-axis represents statistical significance of the enrichment. **(A)** represents the classification of total KEGG, **(B)** represents the top 20 KEGG enriched metabolic pathways in the C2_T6 group, **(C)** represents the top 20 KEGG enriched metabolic pathways in the C2_T12 group and **(D)** represents the top 20 KEGG enriched metabolic pathways in the C2_T24 group.

DEGs were mapped to the KEGG database to further study biological functions and important pathways. The KEGG pathway database was mainly divided into five categories, including metabolism, genetic information processing, environmental information processing, cellular processes and biological systems. Trunk kidney DEGs of channel catfish were placed in 33 functional classifications, while *Y. ruckeri* DEGs were placed in 26 functional classifications (**Figures 5A** and **6A**). Some genes exist in multiple pathways and some were limited to one pathway. The DEGs found in the trunk kidneys of channel catfish were significantly enriched in the functional classifications of signal transmission (1151 genes), followed by the endocrine system (538 genes), immune system (517 genes) and transport and catabolism (431 genes). The main biochemical metabolic and signal transduction pathways involved in DEGs can be determined through pathway significant enrichment. The first 20 most-enriched KEGG pathways of DEGs were noted at 6, 12 and 24 hpi. Systemic lupus erythematosus and alcoholism were significantly enriched at three time points, while the immune related JAK-STAT signaling pathway, viral carcinogenesis and toll-like receiver signaling pathways were significantly enriched at 6 and 12 hpi (**Figures 5B–D**).

DEGs observed in *Y. ruckeri* were significantly enriched for the functional classification of carbon metabolism (152 genes), followed by global and overview maps (140 genes), amino acid metabolism (125 genes) and membrane transport (113 genes). The top 20 most-enriched KEGG pathways of the DEGs were noted at 6, 12 and 24 hpi. There was significantly enrichment in the ribosome and two component systems, but this was only significant at 6 hpi. However, glycospholipid metabolism was also enriched at all points but was not found to be significant only at 6 hpi (**Figures 6B–D**).

### Co-Expression of Host and Pathogen Genes

**Figure 7:**
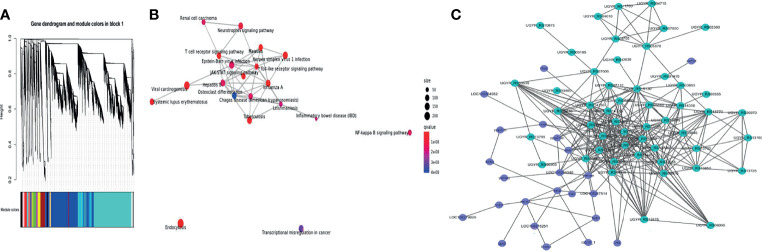
WGCNA of transcriptional changes in channel catfish and Y. ruckeri. **(A)** A time-resolved transcriptome WGCNA cluster map for channel catfish and *Y. ruckeri*. **(B)** KEGG enrichment analysis for the turquoise module. The size of the circle represents the number of genes in the pathway. Color indicates significance and connected pathways represent genes within the pathway. **(C)** A correlation network of metabolic related DEGs in the turquoise module containing channel catfish and *Y. ruckeri* genes with an edge Pearson correlation coefficient greater than 0.98. Dot size represents degree values. Channel catfish genes are shown in purple and Y. ruckeri genes are shown in green.

To determine the co-expression network between channel catfish and *Y. ruckeri* genes during infection, a hierarchical clustering tree was generated for the two organisms using WGCNA (**Figure 7A**). In the same module, the host and pathogen genes co-expressed at different time points were simultaneously identified. A total of 15 samples from 5 groups were used, including the channel catfish and *Y. ruckeri* control group and 3 experimental groups. Each experimental group contained three parallel controls. The gene coexpression network was constructed using all expressed genes. The constructed coexpression modules were divided into 19 different modules and named based on the color of their modules displayed on the hierarchical clustering tree.

To determine the importance of each module, KEGG enrichment analysis was performed. The turquoise module was enriched into many immune related pathways, as shown in **Figure 7B**. Therefore, the turquoise module was considered to be the most important module for the infection process.

To understand the interaction between the host and pathogen during infection, some key genes were selected for analysis. KME values(Pearson correlation coefficient)of genes > 0.98 and edge weights > 0.35 were selected for visualization. The interaction diagram is presented in **Figure 7C**, including 23 genes for the host and 50 genes for the pathogen.

### Verification of DEGs

**Figure 8:**
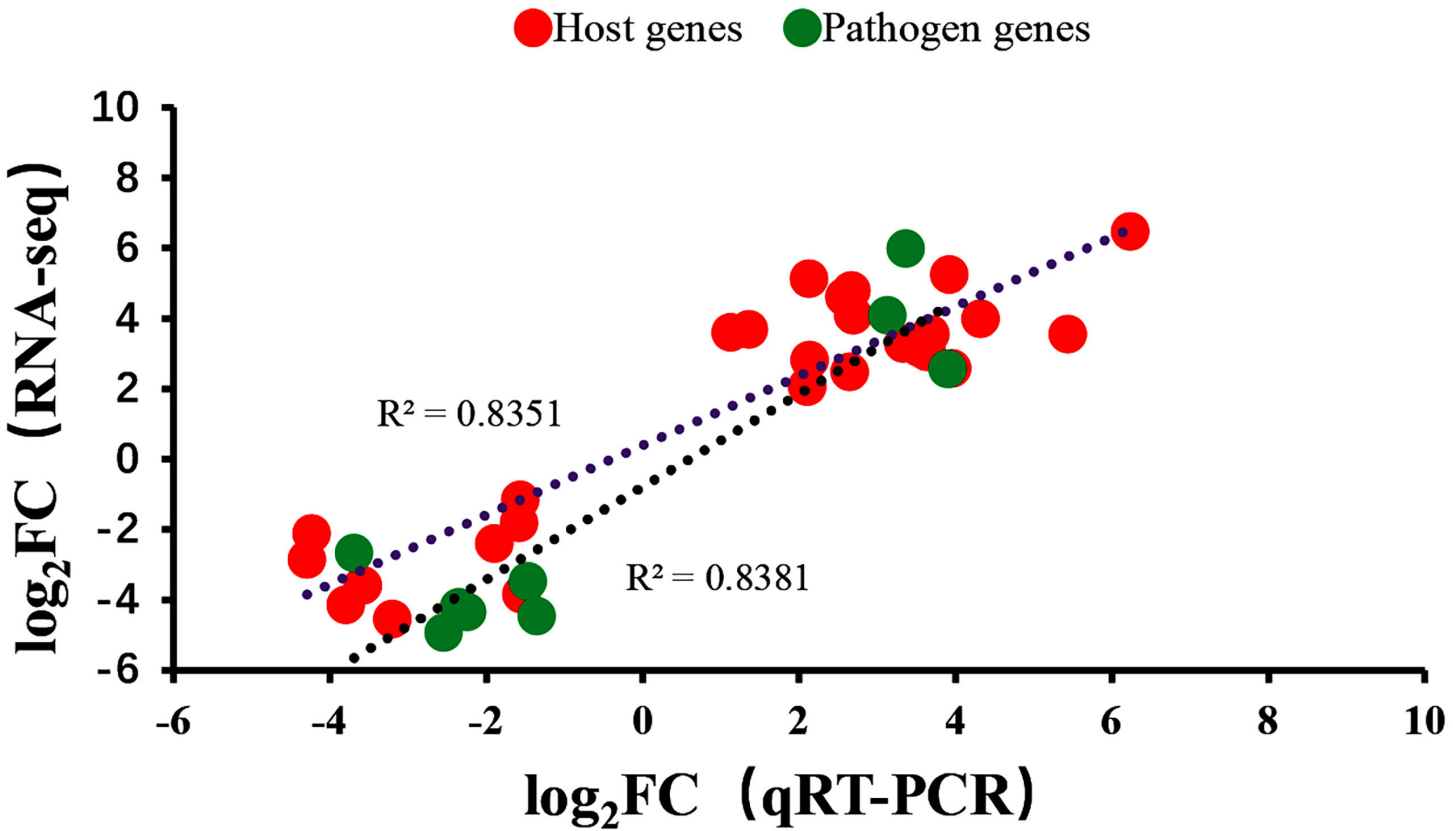
Comparison of gene expression levels for 12 genes using dual RNA-Seq and qRT-PCR. Negative values indicated that the gene expression in channel catfish was downregulated after infection by *Y. ruckeri* and positive values indicated an upregulation in gene expression after infection.

To verify the RNA-seq results, qRT-PCR was performed on the same RNA samples used for sequencing to detect 12 DEGs selected from differential expression data. The primer sequences for all genes are listed in **Table 1**. The qRT-PCR results of the 12 genes analyzed were consistent with results obtained from RNA -seq (**Figure 8**).

## Discussion

*Y. ruckeri* causes ERM in channel catfish and can lead to rapid death of this aquatic species ultimately resulting in heavy economic burdens. Both antibacterial treatment and vaccine prevention are used to control disease caused by *Y. ruckeri* (4, 6, 50, 51). Generally, vaccination is the most effective means of disease prevention in aquaculture. However, this method is very stressful for fish and labor-intensive for farmers (6). Traditionally, the infection rate from bacterial pathogens in aquaculture mainly depended on the use of antibiotics and antibacterial compounds (4). However, due to the high usage of antibiotics, antibiotic resistance and the safety problems related to antibiotic residues are an issue. Therefore, it is necessary to explore the mechanisms behind *Y. ruckeri* invasion of its host and its interaction with a host to provide a theoretical basis for finding gene targets to block bacterial invasion, reduce toxicity and strengthen host immune surveillance.

The interaction between the host and pathogen during infection is complex. During the different stages of infection, transcriptome changes occur in both the host and the pathogen. Not only is trunk kidney an important immune organ, it is also an important excretory organ in fish. At the same time, the trunk kidney is also the main organ infected in *Y. ruckeri* (6). Selecting the trunk kidney as the tissue analyzed during dual RNA-seq sequencing not only reflects the interaction between the host and pathogen, but also detects possible damage to the trunk kidney of channel catfish caused by *Y. ruckeri*, to provide basis for formulating prevention and control measures.

In this study, we investigated transcriptome changes in the trunk kidney and pathogen at different time points after channel catfish inoculation with *Y. ruckeri*. The number of DEGs in the trunk kidney and *Y. ruckeri* changed at the three different time points, but the overall change in the host was small at three time points, where 9000 genes with significant differences were enriched. However, at the 6 hpi point, genes related to innate immunity were enriched, including IL-20-like and IL-1β. There was a significantly higher number of down-regulated genes compared to upregulated genes in the pathogen, and this difference was most obvious at 24 hpi. However, OMPF genes, which are important bacterial antigens, were significantly enriched at all three time points (50). These results indicate that there is a strong immune response between the host and pathogen when *Y. ruckeri* infects channel catfish.

Host innate immunity uses a variety of recognition receptors, such as toll-like receptors, to recognize bacteria (52–54). Transcriptomic analysis of *Oreochromis niloticus* during *Streptococcus agalactiae* infection showed that toll-like receptor-mediated pathways contribute to immune responses and protect the host against pathogens (55). The expression levels of many genes in “toll-like receptor signaling pathway” were significantly upregulated in large yellow croakers infected with *pseudomonas mutants* at 4 dpi (56). The results of this study showed that the expression levels of 58 differential genes in toll-like receptor signaling pathways were significantly upregulated at the onset of infection (6 hpi). Cytokines are key modulators of the host defense in innate immunity and adaptive inflammation (57). Cytokine-cytokine receptor interactions, NF-Kappa B signaling pathways, important innate immune pathways such as the JAK-STAT signaling pathway, and the TNF signaling pathway were significantly enriched at 6-24 hpi, consistent with previous studies (58–61). When these pathways are significantly enriched, the fish produces an acute inflammatory response to protect itself and enhance its tissue repair and defense during infection. These results showed that the immune response of channel catfish increases during the progression of *Y. ruckeri* infection.

Metabolic regulation of the host is also very important in the process of infection. The immune system needs energy to resist pathogens and maintain tissue homeostasis (62, 63). To overcome stress caused by the pathogen, the infected fish expends a large amount of energy (64). Amino acid metabolism is important for host energy consumption, detoxification, protein synthesis and safe operation of innate immune responses (65). Through KEGG enrichment, all pathways enriched at 6-12 hpi are related to immunity. At 24 hpi, many metabolic pathways are enriched, such as fatty acid, glycine, serine and threonine and butanoate metabolism pathways. It is speculated that when channel catfish are infected by *Y. ruckeri*, the acute immune response begins immediately. As the first line barrier, innate immune-related molecules and pathways are rapidly expressed. With more serious and progressed infection, channel catfish require fatty acid and amino acid metabolism to provide energy and proteins. Changes in tryptophan metabolism were also observed in many fish such as zebrafish, large yellow cordon, Atlantic salmon and abalone, indicating that tryptophan and its metabolites may play an important role in the immune system of aquatic animals (66–68).

For pathogens that invade the host, flagella play a key role, including colonizing and invading the host, reaching the optimal host site, maintaining the infection site and spreading after infection (69–71). To date, flagella have been implicated in the virulence of many aquatic pathogenic bacteria, such as *Y. ruckeri* (46, 72), *Edwardsiella tarda* (73), and *Vibrio anguillarum* (74). In this study, the flagellar gene fliC was significantly upregulated from 6-24 hpi, indicating that it was involved in the infection process of *Y. ruckeri* (72). KEGG was significantly enriched in the two-component system of *Y. ruckeri* at 6-12 hpi. This indicates that the bacteria adapt to the state of the fish by sensing environmental changes, regulating the expression of survival and virulence factors and maintaining their own survival (23). At 24 hpi, the gene expression levels of the bacterial two-component system decreased, indicating that the pathogen adapted to the fish environment and the host developed symptoms and began to due. At the same time, the pathogen was significantly enriched in the sucrose of bacterial secretion system, ubiquinone and other terpenoid-quinone biosynthesis, and starch and sucrose metabolism, glycerophospholipid metabolism, one carbon pool by folate and other signaling pathways (75). These results indicated that the pathogen used its secretory system to escape and consume energy during the invasion process.

WGCNA was divided into 19 modules and the results showed that the relationship between the host and pathogen is very complex. The turquoise module was considered to be the most important in the infection process, showing the dynamic process of the life-and-death struggle between the host and the pathogen, reflecting how the innate immunity of the host copes with pathogen invasion and how the pathogen achieves immune escape. In the interaction network diagram, 50 pathogenic genes were found to be associated with the host immune response. The pathogen mainly relied on SpaR and SpaP for immune escape through the type II secretion system, which has not been identified in previous studies (76, 77). The pathogen HlyB was up-regulated in the infection, indicating that the pathogen rely on hemolysin to cause harm in the host trunk kidney. The uvry regulatory gene and flagella transcriptional regulator flhd were also significantly down-regulated, consistent with previous studies that uvry gene mutations did not affect the survival rate of *Y. ruckeri* in fish (23), Therefore, the pathogen mainly depended on fliC flagellin for attachment and virulence. With progression of the infection, the expression of the multidrug resistant proteins mdtc and MDTA were seriously inhibited, suggesting that the drug resistance of the strains decreased.

Here, we successfully used tissue dual RNA-seq to simultaneously analyze the dynamics of gene expression changes in both the host and pathogen and obtained high-resolution transcriptome data. For the host, innate immunity is an important means to resist bacterial invasion and bacterial flagella is an important tool for infection used by the pathogen. WGCNA reveals the strong interaction and relationship between the host and pathogen genomes throughout the infection process, which is most closely related to metabolic activities. This study has important theoretical significance for understanding the pathogenesis of channel catfish ERM.

## Data Availability Statement

The datasets presented in this study can be found in online repositories. The names of the repository/repositories and accession number(s) can be found below: NCBI SRA BioProject, accession no: PRJNA760961.

## Ethics Statement

The animal study was reviewed and approved by Institutional Animal Care and use Committee of Changjiang Fisheries Research Institute, Chinese Academy of Fishery Sciences.

## Author Contributions

YY and YC conceived and designed the study. YY performed most of the experiments. XA, XZ, HZ, and YS provided bioinformatics assistance and support. YC and YY wrote the manuscript. All authors contributed to the article and approved the submitted version.

## Funding

This work was supported by Yancheng fishery high quality development project [No.YCSCYJ2021026], Major innovation projects in Hubei Province [No. 2019ABA077] and the Central Public-interest Scientific Institution Basal Research Fund, CAFS [No.2017HY-ZD0505].

## Conflict of Interest

The authors declare that the research was conducted in the absence of any commercial or financial relationships that could be construed as a potential conflict of interest.

## Publisher’s Note

All claims expressed in this article are solely those of the authors and do not necessarily represent those of their affiliated organizations, or those of the publisher, the editors and the reviewers. Any product that may be evaluated in this article, or claim that may be made by its manufacturer, is not guaranteed or endorsed by the publisher.
