# Corrigendum: Dual RNA-seq of trunk kidneys extracted from channel catfish infected with *Yersinia ruckeri* reveals novel insights into host-pathogen interactions

**DOI:** 10.3389/fimmu.2022.982091

**Published:** 2022-07-27

**Authors:** Yibin Yang, Xia Zhu, Haixin Zhang, Yuhua Chen, Yi Song, Xiaohui Ai

**Affiliations:** ^1^Yangtze River Fisheries Research Institute, Chinese Academy of Fishery Sciences, Wuhan, China; ^2^The Key Laboratory for Quality and Safety Control of Aquatic Products, Ministry of Agriculture, Beijing, China; ^3^Fish Disease Laboratory, Jiangxi Fisheries Research Institute, Nanchang, China; ^4^Department of Gastroenterology, Zhongnan Hospital of Wuhan University, Wuhan, China; ^5^Hubei Clinical Center & Key Lab of Intestinal & Colorectal Diseases, Zhongnan Hospital of Wuhan University, Wuhan, China

**Keywords:** channel catfish, *yersinia ruckeri*, dual RNA-seq, host-pathogen interactions, immunity, virulence

In the published article, there was an error in **Figure 6A** as published. **Figure 5A** was used instead of **Figure 6A** in the revision of the article. The corrected **Figure 6A** appears below.

**Figure 6:**
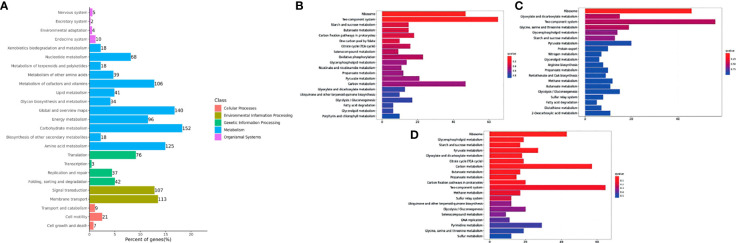
Histogram of the top 20 enriched KEGG pathways of DEGs in *Y. ruckeri* at different time points. The Y-axis represents the KEGG pathway categories. The X-axis represents statistical significance of the enrichment. **(A)** represents the classification of total KEGG, **(B)** represents the top 20 KEGG pathways in the C2_T6 group, **(C)** represents the top 20 KEGG pathways in the C2_T12 group and **(D)** represents the top 20 KEGG pathways in the C2_T24 group.

In the published article, there was an error in the legend of **Figure 1** as published. There was one mistake in the species names. The corrected legend appears below.

**Figure 1:**
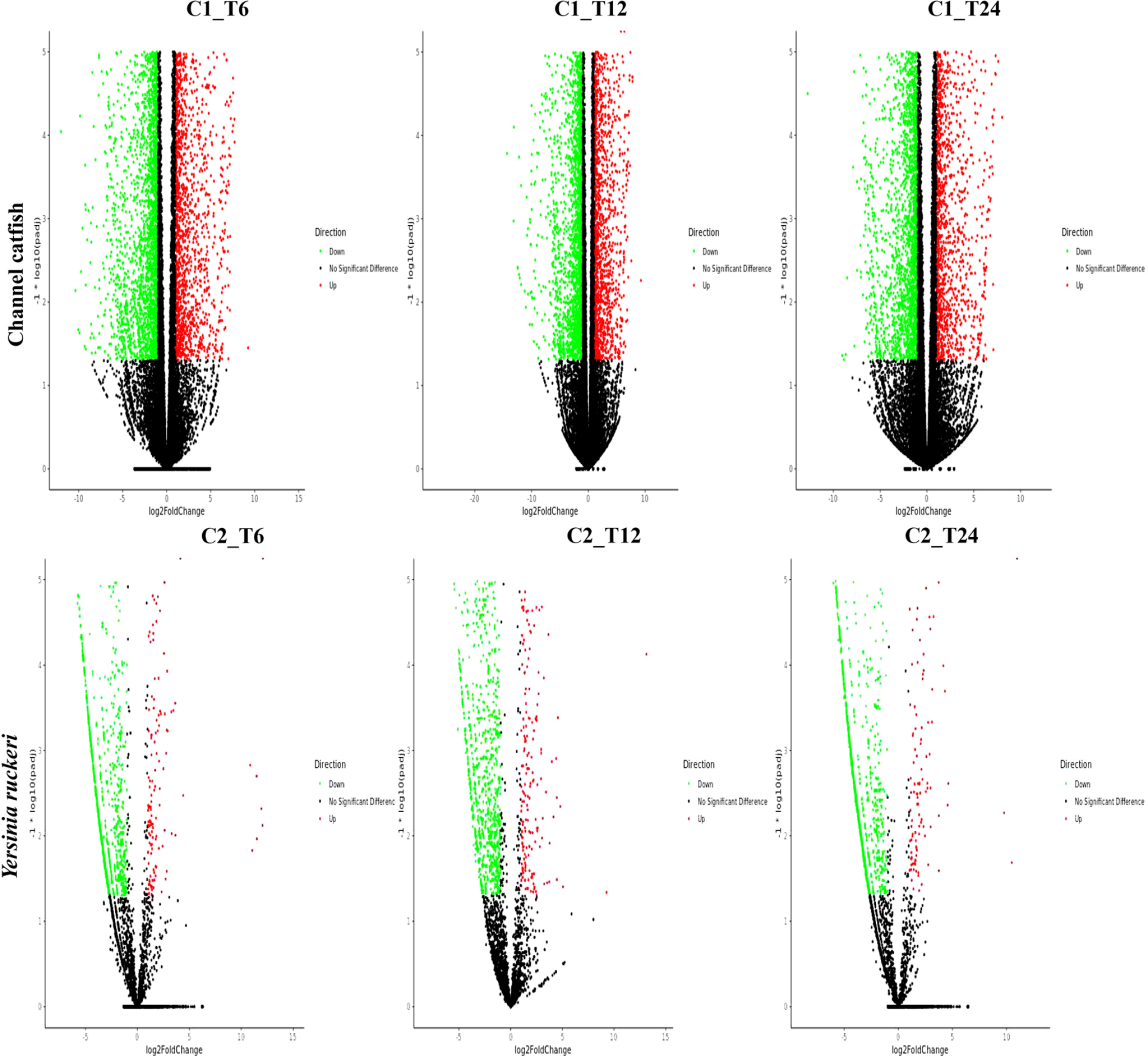
The gene expression profiles for channel catfish and *Y. ruckeri* during infection. A volcanic plot depicting the differences in the expression profiles of channel catfish and *Y. ruckeri*. The X-axis represents a log2 (fold change) and the Y-axis represents -log10 (p value). Red represents significantly upregulated genes whereas green represents significantly downregulated genes. Each dot represents a single gene. C1_T6(C2_T6) represents 6 hpi, C1_T12(C2_T12) represents the 12 hpi and C1_T24 (C2_T24) stands for 24 hpi.

“**Figure 1**. The gene expression profiles for channel catfish and *Y. ruckeri* during infection. A volcanic plot depicting the differences in the expression profiles of channel catfish and *Y. ruckeri*. The X-axis represents a log2 (fold change) and the Y-axis represents -log10 (p value). Red represents significantly upregulated genes whereas green represents significantly downregulated genes. Each dot represents a single gene. C1_T6(C2_T6) represents 6 hpi, C1_T12(C2_T12) represents the 12 hpi and C1_T24(C2_T24) stands for 24 hpi.”

In the published article, there was an error. A correction has been made to **DISCUSSION**, Paragraph 7. This sentence previously stated:

“The pathogen HlyA was severely down-regulated by the host, indicating that the pathogen did not rely on hemolysin to cause harm in the host trunk kidney.”

The corrected sentence appears below:

“The pathogen HlyB was up-regulated in the infection, indicating that the pathogen rely on hemolysin to cause harm in the host trunk kidney.”

The authors apologize for these errors and state that they do not change the scientific conclusions of the article in any way. The original article has been updated.

## Publisher’s note

All claims expressed in this article are solely those of the authors and do not necessarily represent those of their affiliated organizations, or those of the publisher, the editors and the reviewers. Any product that may be evaluated in this article, or claim that may be made by its manufacturer, is not guaranteed or endorsed by the publisher.

